# Roles of Chromatin Remodelling and Molecular Heterogeneity in Therapy Resistance in Glioblastoma

**DOI:** 10.3390/cancers14194942

**Published:** 2022-10-09

**Authors:** Huey-Miin Chen, Ana Nikolic, Divya Singhal, Marco Gallo

**Affiliations:** Arnie Charbonneau Cancer Institute, Alberta Children’s Hospital Research Institute, Cumming School of Medicine, University of Calgary, Calgary, AB T2N 4N1, Canada

**Keywords:** glioblastoma, epigenetics, chromatin, therapy resistance, epidrugs

## Abstract

**Simple Summary:**

We review the role of chromatin and epigenetic dysregulation in therapy resistance in glioblastoma. We discuss how epigenetic and genetic forces may cooperate to programme functional cell states that are inherently resistant to therapy. Targeting epigenetic factors that are dysregulated in this malignancy could, therefore, improve clinical outcomes for patients. We highlight some preclinical and clinical compounds that were tested or are currently being explored for glioblastoma. Lastly, we present our thoughts on the requirements for the development of next-generation epigenetic therapies.

**Abstract:**

Cancer stem cells (CSCs) represent a therapy-resistant reservoir in glioblastoma (GBM). It is now becoming clear that epigenetic and chromatin remodelling programs link the stemlike behaviour of CSCs to their treatment resistance. New evidence indicates that the epigenome of GBM cells is shaped by intrinsic and extrinsic factors, including their genetic makeup, their interactions and communication with other neoplastic and non-neoplastic cells, including immune cells, and their metabolic niche. In this review, we explore how all these factors contribute to epigenomic heterogeneity in a tumour and the selection of therapy-resistant cells. Lastly, we discuss current and emerging experimental platforms aimed at precisely understanding the epigenetic mechanisms of therapy resistance that ultimately lead to tumour relapse. Given the growing arsenal of drugs that target epigenetic enzymes, our review addresses promising preclinical and clinical applications of epidrugs to treat GBM, and possible mechanisms of resistance that need to be overcome.

## 1. Introduction

Glioblastoma (GBM) is the most prevalent malignant brain tumour in the adult population. Despite significant advances in dissecting the genomic, epigenomic, and molecular factors that drive GBM aetiology, this malignancy is still incurable, and patients have dismal prognoses. In this review, we discuss the notion that epigenetic principles play pivotal roles in GBM. Therefore, therapies that target epigenetic mechanisms have strong potential to become curative or at least improve the current standard of care for this cancer. We discuss the rationale for epigenetic therapy in GBM and promising preclinical data, and assess some approaches that are being tested in clinical trials. Lastly, we provide an outlook for future consideration of epigenetic therapy for this lethal cancer.

## 2. Cancer Stem Cells Represent Reservoirs of Therapy Resistance in GBM

Chromatin is composed of genomic DNA and its interacting proteins. In contrast to nucleotide sequences, chromatin structures are highly dynamic and support the spatiotemporal regulation of gene expression without alteration to the primary sequences [1]. Major epigenetic features that reshape chromatin structures include the acetylation or methylation of the N-terminal histone tail [2], the methylation of the DNA cytosine at position 5 on the pyrimidine ring [3], and the noncoding RNA-controlled pre- and post-transcriptional regulation of gene expression [4]. Interestingly, genes involved in chromatin remodelling are frequently mutated or dysregulated in GBM [5,6,7,8]. Here, we present a review of mechanisms by which the impairment of chromatin remodelling machinery can confer therapeutic resistance in GBM.

Chromatin remodelling is coupled with epigenetic modifications. During tumourigenesis, dynamic changes to epigenetic factors can result in permissive chromatin states that promote the generation of cancer stem cells (CSCs). The CSC paradigm postulates that tumour cells can be organized in a hierarchical fashion, comprising mostly cells with limited proliferative potential and including a subset of cells at the apex of the hierarchy that exhibits self-renewal capacity [9]. In the early 2000s, Singh and colleagues isolated a subpopulation of tumour cells from dissociated GBM samples that demonstrated capacity to extensively self-renew and proliferate [10,11]. This cell fraction propagated tumours upon xenotransplantation [11], thus establishing GBM-CSCs or glioma stemlike cells (GSCs) as a basis of tumourigenesis and tumour propagation. 

**Figure 1:**
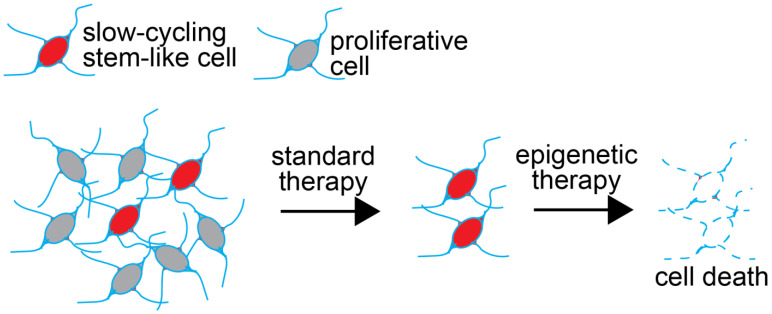
Strategy to deploy epigenetic therapy to target cancer stem cells. Standard therapy (e.g., radiation and most chemotherapies) often consists of treatments that aim to kill actively proliferating cells. These therapies may not eradicate the cancer stem cell population because of its slow cycling behaviour. Combining standard of care approaches with epidrugs designed to target essential epigenetic programs in cancer stem cells could result in the demise of this refractory cell population. This could be achieved in a proliferation-independent way.

Work from the past decade has clearly demonstrated that GSCs are therapy-resistant. Standard treatments of GBM include maximal resection followed by concomitant and adjuvant temozolomide to radiotherapy, also known as the Stupp protocol [12]. While short-term survival has improved since the landmark 2005 Stupp trial, augmentation to longer-term survival (e.g., 5-year) has yet to be realized, and GBM remains an incurable disease [13]. GSCs confer tumour resistance to radio-/chemotherapies by way of their heightened capacity for DNA repair and mitochondrial reserve [14,15], their location in hypoxic niches [16,17], and, most notably, their ability to enter a state of quiescence that renders GSCs refractory to therapies that target actively dividing cells [18,19,20]. New therapies that target cells irrespective of their cycling status are required to eliminate GSCs (Figure 1). In this review, we discuss the epigenetic dependencies of GSCs and the promise of epigenetic therapy to achieve their demise.

## 3. Chromatin Dynamics Contribute to the Therapy-Resistance Properties of GSCs

Cellular quiescence is a reversible state of resting at G_0_ phase [21]. Studies in neural stem cells (NSCs) demonstrated the essentiality of stem cell quiescence in maintaining the stem cell pool for tissue homeostasis and regeneration [22,23,24,25]. This nondividing state is associated with histone modifications that keep genes in a transcriptionally ‘poised’ state, consequently maintaining quiescent stem cells in readiness for rapid cell cycle re-entry, self-renewal, and differentiation [26]. In embryonic stem cells (ESCs), key developmental genes are associated with bivalent histone domains, e.g., regions with concurrent presence of antagonistic methylation marks on H3 lysine 4 (H3K4me3, activating) and H3 lysine 27 (H3K27me3, repressing) [27]. These bivalent domains are resolved quickly upon differentiation, and poised genes are either activated by the removal of H3K27me3 or repressed by loss of H3K4me3 [27,28]. Adult stem cells, such as hematopoietic stem cells (HSCs), demonstrate the presence of bivalent chromatin domains. In quiescent HSCs, the resolution of bivalent domains results in gene expression changes that stipulate lineage commitment [29]. Notably, the single-cell RNA-seq of GBM tumour specimens identified a population of slow-cycling quiescent GSCs that showed a significantly higher expression of *KDM5B* [30]. Moreover, upon the inhibition of proliferative receptor tyrosine kinase (RTK) signalling, GSCs can transition into a KDM6-dependent slow-cycling cellular state that would render GSCs refractory to standard therapies [31]. As KDM5B and KDM6(A/B) resolve bivalency at developmental genes via H3K4 and H3K27 demethylation, respectively [32,33,34], chromatin remodelling by way of histone (de)methylation at bivalent domains is likely to facilitate the hijacking of developmental gene expression programmes to generate treatment-resistant quiescent GSCs. Indeed, the mapping of GBM scRNA-seq data onto a detailed atlas of mouse cerebral development revealed the acquisition of embryonic precursor identities in the genesis of glioma [35]. Ube2V2, a ubiquitin-conjugating protein, was the top common gene product shared between GBM samples and embryonic radial glial precursors [35]. Ube2V2 regulates H4K16 acetylation [36], a histone modification that is associated with HSC quiescence [37]. H4K16ac also forms a *trans-*histone module with H3K4me3 that is recognized by the chromatin remodeller BPTF [38]. Considering that BPTF regulates stem cell maintenance in HSCs [39], quiescent/proliferative states transition into melanoma [40], and the maintenance of self-renewal in GBM [41], and the concurrence of H3K4me3 and H4K16ac histone marks may play a key regulatory role in GSC quiescence. While associations between other histone modifications and cellular dormancy were established in various quiescence model systems, most were performed outside the context of GSCs [26]. Nevertheless, the knockdown of the H3K9me3 histone demethylase KDM4C appears to obstruct the upkeep of GBM stemlike cell lines [42,43], suggesting a role for H3K9 methylation in GSC maintenance and thus therapy resistance.

The initiation and maintenance of changes in gene expression that are associated with stem cell quiescence and self-renewal involve the coordinated action of multiple epigenetic programmes. A deeper understanding of how GSCs restructure their epigenome to enter/exit quiescence opens novel therapeutic avenues for GBM. Rapidly advancing single-cell genomewide technologies and machine-learning capabilities may soon lead to the identification of specific DNA loci and chromatin structures that underly GSC quiescence.

## 4. DNA and Histone Methylation Regulate Self-Renewal Pathways

Alterations in DNA methylation patterns are loosely associated with cellular quiescence. One of the most common somatic mutations occurring in glioma involve isocitrate dehydrogenase (IDH) enzymes [44], which are associated with reduced levels of α-ketoglutarate (α-KG) caused by the conversion of α-KG into D-2-hydroglutarate (2-HG) [45,46]. Multiple histone and DNA demethylases utilize α-KG as an essential cofactor [47,48]. Whereas the addition of α-KG to naïve-state mouse embryonic stem cells (mESCs) induced H3K27me3 demethylation and enhanced self-renewal [49], in primed human pluripotent stem cells (hPSCs) and mouse epiblast stem cells (EpiSCs), α-KG accelerated differentiation via the induction of global histone and DNA demethylation [50]. Moreover, the competitive inhibition of histone demethylases KDM4A/C by 2-HG impairs cellular differentiation [51,52]. Interestingly, the production of 2-HG upon the activation of transcription factor FoxO1 was crucial for the acquisition of a quiescent endothelial state [53]. FoxO transcription factors are a major point of convergence of RTK signalling pathways, and enforce NSC quiescence [54]. Enhanced DNA methylation at the promoter region of *FoxO3* promotes GSC self-renewal [55]. Nonetheless, while direct links between α-KG-dependent demethylation and enhanced cellular heterogeneity were demonstrated recently in breast cancer [56], we have yet to discern the direct impact that altered DNA methylation may have on GSC cellular heterogeneity or quiescence. 

## 5. Epigenetic Roles of Long Noncoding RNAs in GBM

In addition to histone modifications and DNA methylations, long noncoding RNAs (lncRNAs, >200 nt) have emerged as important players in chromatin remodelling [57,58,59]. A well-established attribute of lncRNAs is their bridging of chromatin modification complexes to DNA. For example, the lncRNA *Xist* interacts with polycomb repressive complex 2 (PRC2) member EZH2 to mediate H3K27 trimethylation of the inactive X chromosome [60]. *HOTAIR*, a lncRNA transcribed from the HoxC locus, recruits PRC2 to the HoxD cluster to facilitate gene silencing [61]. The lncRNA *H19*, a regulator of HSC quiescence [62], can alter H3K9me3 histone modifications by its binding to MBD1, a methyl-CpG binding protein [63]. Meanwhile, *Xist*, *HOTAIR* and *H19* were all linked to glioma angiogenesis and/or GBM cell cycle progression [64,65,66]. The association of lncRNAs with GBM was comprehensively surveyed in a recent review by Yadav and colleagues [67]. Still, much remains to be discovered with regards to the role that lncRNAs play in GSC maintenance. 

## 6. Intratumoural Epigenetic Heterogeneity and Its Role in Drug Resistance

Intratumoural heterogeneity refers to the coexistence of genetically, epigenetically, and functionally different cell subpopulations within a single tumour. This diversity of cell populations might contribute to treatment failure by providing alternative pathways of therapy resistance. On the molecular level, this phenomenon is represented by disparate subclonal mutations and structural variants and gene expression patterns [5,8,68,69,70]. Intratumoural heterogeneity can be assessed in several ways: classically, by multiregional sampling or sampling at different points in time, and more recently, by sampling at a single timepoint using technologies such as single-cell sequencing.

As examples of genetic heterogeneity contributing to therapy resistance, multiple RTK genes such as *EGFR*, *PDGRA* and *MET* are usually amplified in mutually exclusive fashion in genetic subclones [71,72,73]. It is, therefore, conceivable that patients treated with EGFR inhibitors, for instance, have treatment-resistant genetic subclones represented by the *PDGFRA*-amplified cells. The use of single-cell technologies to test the molecular architecture of subclones could, therefore, be used to design more appropriate treatment cocktails that account for potential resistance mechanisms.

The development of single-cell technologies has also aided in the characterization of transcriptional heterogeneity in gliomas. In 2014, Patel and colleagues profiled cells from five GBM samples with scRNA-seq and found individual cells in all tumours that matched at least two of the TCGA molecular subtypes [30]. Interestingly, the authors found that tumours with more transcriptional diversity also had worse prognosis than that of tumours with lower levels of transcriptional heterogeneity. This example underscores the importance of intratumoural heterogeneity in tumour aggressiveness, potentially through cooperation between the different cell states present in a tumour.

Tumour heterogeneity in GBM may also arise from clonal evolution, that is, through the expansion of treatment-resistant GSCs and the acquisition of mutations during tumour progression that promote genetic variability [74]. This concept may be linked to the hierarchical CSC model where a small subpopulation of stemlike cells, maintained through their ability to self-renew, facilitate the production of diverse daughter cells that repopulate the tumour bulk [75]. These cell state changes may be driven by factors in the tumour microenvironment and epigenetic aberrations within the tumour itself.

Several cancer models have cell state transitions or transient epigenetic changes that allow for tumour cells to persist through drug exposure [31,76,77]. The comprehensive assessment of pre-existing and dynamic genetic and epigenetic drug resistance mechanisms remains a significant goal that requires attention. In a recent study, Bernstein’s group developed strategies combining scRNA-seq and lineage tracing to evaluate the interplay between genetic and epigenetic mechanisms of resistance in stemlike GBM cells treated with RTK inhibitors [78]. They showed that genetic subclones bearing insulin receptor substrate-1 (*IRS1*) amplifications exhibit a degree of reversibility to dasatinib resistance that may underlie the inefficacy of targeted therapies. 

**Figure 2:**
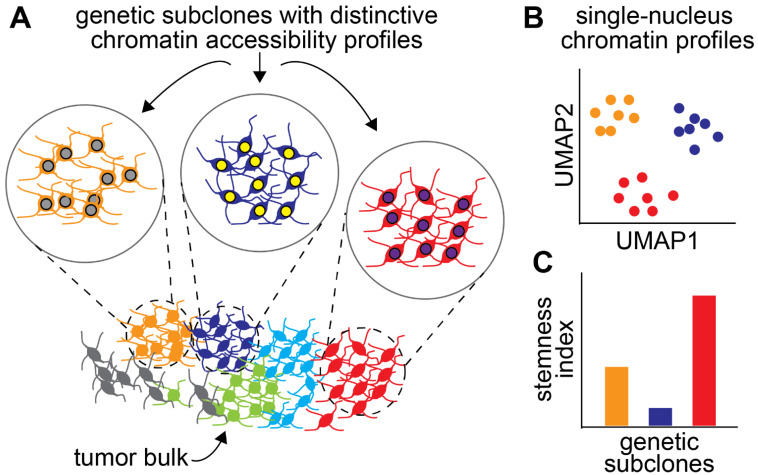
Genetic subclones can have characteristic chromatin landscapes. (**A**) Genetic subclones coexist in a tumour. Each colour marks cells in a subclone. Subclones can have distinctive epigenetic profiles, represented by the colour of the nuclei. (**B**) Single-cell or single-nucleus approaches such as ATAC-seq can reveal the differences in chromatin profiles between genetic subclones. (**C**) Because of their specific chromatin landscape, each subclone can be predisposed to behave like cancer stem cells or not.

It is now becoming clear that genetic, epigenetic, and functional heterogeneities are interdependent. For instance, Neftel et al. showed that transcriptional states in GBM are associated with specific mutational profiles [79]. Similarly, genetic subclones have unique chromatin accessibility profiles in GBM (Figure 2A,B) [80]. These studies echo earlier work by the Costello laboratory showing the parallel evolution of genetic and DNA methylation/epigenetic landscapes in gliomas [81]. Given the dependence of GSC properties on epigenetic and transcriptional states, it is reasonable to speculate that some genetic subclones may be more predisposed to give rise to stemlike cells than others are (Figure 2C). This hypothesis is supported by the enrichment of motifs for transcription factors associated with stemness in the accessible chromatin of some subclones [80]. These observations could help in prioritizing the targeting of genetic subclones in primary tumours.

## 7. Epigenetics, Plasticity, and the Tumour Microenvironment

Single-cell studies have enabled a more granular view of the tumour cell state and epigenetic heterogeneity in high-grade glioma. GBM cells have a characteristic distribution in the tumour microenvironment, with stemlike cells being enriched in the perivascular niche [82], mesenchymal-like cells expressing the transcription factors MLL1 and HIF2a and associated with more hypoxic areas of the tumour microenvironment [17,83,84], and SOX10-positive oligodendrocyte-like cells preferentially existing in the subcortical white matter [85]. However, the extent to which these cellular states are governed by tumour genetics versus epigenetics is unclear.

Recent DNA methylation studies have highlighted the importance of methylation in mediating tumour plasticity in response to environmental stimuli in IDH wild-type (IDHwt) and mutant (IDHmt) gliomas with higher DNA methylation disorder associated with stress-response genes, which was more pronounced after hypoxia and associated with cell state transitions [86]. The spatial RNA sequencing of 28 GBM specimens described five distinct subtypes partially overlapping with previously described cellular states. Neuronal development-like and reactive-hypoxia like cells were less proliferative. Moreover, most genetic subclones contained multiple transcriptional states, highlighting the importance of epigenetic regulation. The exception was the reactive-hypoxia state, which was associated with increased genomic instability [87]. Longitudinal profiling shows increased mesenchymal state enrichment upon recurrence in IDHwt glioblastoma, with some association to *NF1* loss of function [88]. The profiling of paired recurrent IDHwt and IDHmt gliomas showed transitions to mesenchymal states in a large proportion of IDHwt tumours, and enriched neuronal signalling in another subset, again with few associated genetic changes [89]. Overall, this suggests that, while genetics may play a modifying role, epigenetics is likely a primary driver of cellular state heterogeneity in GBM.

Epigenetic alterations may also be important regulators of tumour-immune cell relationships, especially in the context of mesenchymal-type GBM. These tumours have a highly immunosuppressive microenvironment, enriched for M2 macrophages (reviewed in [90]). This is partly due to migration of peripheral macrophages to tumours driven by secreted factors such as periostin (POSTN) [91]. This transcriptional state in human and mouse is partially driven by macrophage-driven Osm secretion, which remodels both tumour cell and macrophage transcriptional profiles, suggesting a bidirectional interaction between the tumour and the infiltrating immune cells [92]. The single-cell profiling of T-cell repertoires across a number of gliomas identified an NK-cell like subset of cytotoxic T cells marked by CD161 with increased PDCD1 activity and the impaired killing of tumour cells, highlighting the bidirectional interplay between tumour and immune cells [93]. The profiling of IDHmt gliomas by single-cell ATAC-seq identified *ATRX* loss as a positive regulator of chemotaxis factors like CSF1. *Atrx* knockout in mice resulted in an immunosuppressive tumour microenvironment and a more astrocyte-like phenotype driven by NFKB1 [94]. The serial transplantation of genetically engineered mouse glioma models in immunocompetent mice resulted in epigenetic remodelling that lead to the increased secretion of chemokines such as Ccl9 and Irf8, and a mesenchymal-like tumour state with increased expression of myeloid genes. Interestingly, this behaviour was dependent on microenvironmental macrophages [95]. This may partly be mediated by Notch signalling as, in mouse gliomas, the knockout of Rbpj is associated with more proliferative tumours, increased macrophage infiltration and impaired T-cell response [96]. Indeed, epigenetic remodelling in GBM may be bidirectional, with the involvement of both tumour and immune cells, and this may pose an important caveat for future epidrug treatments. 

GBM cells also have more direct interactions with their microenvironment, which can modulate tumourigenicity and aggressiveness. In paediatric gliomas, the neuronal secretion of neuroligin-3 stimulates tumour growth, and its blockade via the inhibition of the ADAM10 sheddase markedly inhibits tumour growth [97,98]. Gliomas also form direct tumour-promoting synapses with neurons in both adult and paediatric gliomas through AMPA-receptor mediated circuits, and the blockade of these circuits reduces the invasiveness of microtube-bearing cells and tumour growth [99,100]. Intercellular communication in GBM is critically important, and its downstream epigenetic mechanisms are yet largely unexplored. 

## 8. Targeting the Epigenetic and Chromatin Factors in GBM

Aberrant epigenetic and chromatin mechanisms are a clear feature of GBM and particularly of CSCs. The genomic analysis of a cohort of adult GBM samples collected by The Cancer Genome Atlas (TCGA) showed that approximately half of all cases had at least one mutation in genes encoding chromatin or epigenetic factors [8]. This observation suggests that tumour cells optimize their epigenome to maximize their malignant behaviour, with mutational signatures reflecting that some epigenetic and chromatin processes promote tumourigenesis, and others likely antagonize tumourigenesis by encoding developmental differentiation pathways. In addition, some mutations confer neomorphic functions to the encoded gene product that can contribute to epigenetic states that are optimal for tumour growth, as was observed for mutations in *IDH1*/2 and histone 3 [7,44]. At least some of these mutated epigenetic pathways directly contribute to the stemness properties of malignant cells [101]. 

However, a second tier of epigenetic and chromatin mechanisms are co-opted directly by the CSC populations in GBM to further potentiate their stemlike phenotypes. This second tier of epigenetic mechanisms is not necessarily co-opted through mutational inactivation or gain of function, but through transcriptional dysregulation. Because transcription is itself dependent on epigenetic regulation, CSC properties might depend on the maintenance of epigenetic-transcriptional feedback loops that are optimal for the execution of their malignant functions. Some of these loops might ultimately depend on clonal or subclonal mutations in genes encoding epigenetic and chromatin factors [80].

Because of the importance of epigenetic mechanisms for GBM aetiology and specifically for the maintenance of CSC populations (as described earlier in this review), pharmaceutically targeting epigenetic and chromatin factors could be a promising therapeutic avenue. Over the past decade, a large arsenal of such drugs, often referred to as ‘epidrugs’ because of their targets, have been designed [102,103]. Largely, epidrugs are tool compounds, but some have performed well in preclinical assays, and a few have been deployed in clinical trials and are discussed in more detail below. 

Epidrugs have to overcome significant hurdles to be effective. First, they need to target functionally important protein domains. This is not always easy because chromatin remodellers and other epigenetic factors are often part of large complexes, and multiple domains are concomitantly involved in protein–protein interactions. In this context, identifying an important domain that is available to bind to a chemical compound is not trivial. This is also true for transcription factors, which can be promiscuous and play multiple roles in transcriptional regulation depending on with which partners they are interacting and the genomic context of their targets. Furthermore, some TFs have intrinsically disordered domains [104] that are functionally important but very difficult to target because of their lack of structure. Second, some epidrugs have to be compatible with principles of liquid–liquid phase separation (LLPS) to achieve therapeutically relevant concentrations inside the nucleus (reviewed in [105]). This point is best exemplified by BRD4 inhibitor JQ1. Recent work from multiple laboratories showed that BRD4 participates in the formation of condensates that separate from the rest of the nucleus through principles of LLPS [106,107]. BRD4 inhibitor JQ1 concentrates in LLPS condensates formed by BRD4 [108]. Similarly, commonly used chemotherapeutic agents, including cisplatin, concentrate in condensates formed by the mediator complex [108]. Therefore, the ability of some drugs to reach adequate concentrations to achieve therapeutic windows depends on their propensities to participate in liquid condensates. Although LLPS is not unique to the nucleus, understanding how drugs segregate in condensates to interact with their molecular targets, and how to improve this segregation, is very important for epidrugs, given that liquid condensates appear to drive various aspects of the function of nuclear proteins. The problem of drug compatibility with the condensate organization of nuclear proteins has received new impetus because of recent, groundbreaking work in the nuclear LLPS field. Epidrug design should, therefore, be optimized for (i) binding site specificity on the protein target, (ii) the functional promiscuity of the target, and (iii) compatibility with the biology of LLPS condensates.

In addition, epidrugs should be designed to overcome the blood–brain barrier, although this is a problem shared with all drugs that need to reach brain malignancies. However, this problem might be mitigated by recent advances in drug delivery to the brain (for a recent review, see [109]). This topic is outside of the scope of this review, but it should be considered when evaluating drugs for preclinical and clinical trials.

## 9. Epidrugs with Promising Action in Preclinical Models

Following the elucidation of chromatin and epigenetic factors that are important for GBM and specifically CSC function, some epidrugs were successfully deployed in preclinical models. Several inhibitors of polycomb repressive complex 2 (PRC2) function have shown activity in patient-derived models in vitro [110]. A few chemical compounds that target PRC2 and specifically EZH2, which is the enzymatic subunit of this complex, have been tested more prominently. These are UNC1999 and GSK343. Surprisingly, although biomolecular approaches have clearly established the importance of EZH2 for GBM CSC function, EZH2 inhibitors have been tested in very few high-quality preclinical models of adult GBM. Data exist on the effectiveness of these inhibitors on in vivo xenografts generated from IDH1-mutant gliomas and on xenografts generated from paediatric high-grade glioma samples [111]. The latter example established the preclinical efficacy of tazemetostat, an FDA-approved EZH2 inhibitor. Besides this work, a significant number of publications have contributed to the notion that EZH2 inhibitors are effective but poor models of GBM, including cell line U87 (see, for instance, [112,113]), which recapitulates salient features of GBM, including invasion into the brain tissue upon transplantation in mouse recipients. Other publications used DZNeP [114,115], which is more generally a methyltransferase inhibitor that is not specific to PRC2; therefore, conclusions reached with this compound should be carefully evaluated. 

Inhibitors of DNA methylation, for example, 5-azacytidine and decitabine, intercalate in the DNA double helix and block the function of DNA methyl transferases. Both DNA demethylating agents mentioned above are FDA-approved for the treatment of myelodysplastic syndromes and some leukaemias, and may represent a new and effective class of epigenetic therapies (for a review, see [116]). One of the reasons for optimism in the use of the compounds was the recent discovery that these they can induce viral mimicry, a type of cell-intrinsic immune surveillance program meant to protect a cell from viral attack [117,118]. DNA demethylating agents cause the demethylation of repetitive regions, including transposable elements, which are then transcribed to produce double-stranded RNA that is recognized as a foreign entity by the cell machinery and triggers an interferon-based antiviral response [119]. Some evidence of the efficacy of DNA demethylating agents in GBM exists, but it is mostly based on poor models of this tumour type. DNA methylation inhibitors showed promise in xenografts derived from IDH1-mutant gliomas [120]. The success of this approach in a preclinical setting was ascribed to IDH1-mutant gliomas having a CpG island methylator phenotype (CIMP) characterized by the robust and widespread methylation of CpG islands [121]. The jury is still out on the use of a DNA demethylating agent to treat GBM patients, partly because of the reported potential side effects of these compounds [122]. 

The MLL complex is also important for the maintenance of self-renewal in GSCs [83,123]. Several approaches were taken to target this complex. The MLL complex is rather large, and includes many proteins required for the docking of catalytic subunit MLL1 to chromatin. One strategy to compromise the function of this complex was to design various compounds that prevent proper docking of the complex. A critical subunit in this respect is WDR5 [124,125], which is essential for the sustained methyltransferase activity of MLL1. Several WDR5 inhibitors were tested in GBM. A very recent preprint from the Lathia laboratory showed the efficacy of this approach against GBM CSCs in vitro and in flank xenograft models [126]. Another critical subunit of the MLL complex is menin, which is essential for docking of the complex to chromatin [127]. Several menin inhibitors have been developed, including by the Cierpicki, Grembecka, and Armstrong laboratories [128,129,130]. Some of these are tool compounds, but two of them were developed into clinical compounds that specifically target the interactions between menin and MLL fusion proteins, which are oncogenic drivers in a subtype of leukaemia. The tool compounds were tested in patient-derived GBM models, and showed potent efficacy at both reducing viability and self-renewal of GSCs [131,132]. These results highlight the potential usefulness of menin inhibitors in the context of GBM, even in the absence of MLL fusions in this disease. Designing new menin inhibitors that are agnostic to MLL fusion status and that can cross the blood–brain barrier could be a new avenue to explore with broad utility not just to GBM, but likely other brain malignancies as well.

MLL fusion proteins recruit DOT1L to chromatin. DOT1L is the only known H3K79 methyltransferase, and its function facilitates transcription initiation (reviewed in [133]). CRISPR screens showed that DOT1L is essential for GSC viability and stemness function [134,135]. DOT1L inhibitors are effective at suppressing self-renewal and stemness features of GSCs and lead to differentiation phenotypes [135]. However, the assessment of DOT1L inhibitors with orthotopic patient-derived xenografts and direct drug administration in animals is still missing. 

A summary of the epigenetic drugs and their targets discussed in this section are summarized in Table 1.

## 10. Mechanisms of Resistance to Epigenetic Therapies

The current standard of care for GBM patients includes maximal safe surgical resection followed by concomitant radio- and chemotherapy, the so-called Stupp method [12,136]. The used chemotherapy is temozolomide based on clinical data showing a small but statistically significant improvement in patient survival when this drug is added to radiation. However, temozolomide treatment for GBM patients should be considered as palliative care, because the tumours inevitably recur within 4–6 months. Furthermore, temozolomide is an alkylating agent that can cause high mutational burdens in recurring tumours [70], with the consequent effect of speeding tumour evolution and, in some cases, rendering the tumour more aggressive. 

It is reasonable to hypothesize that epidrug-based treatment should not drastically increase the mutational burden of a tumour, at least for the drugs that do not directly affect chemical modifications of DNA or chromatin proteins also involved in DNA damage response. In this respect, therefore, epidrugs could have fewer unintended consequences on tumour evolution. However, epidrugs could push tumour cells to undergo “epigenetic evolution,” forcing cells to readjust their epigenomes to compensate for the biological effects of the treatment. Epidrug-induced epigenetic evolution could result in novel mechanisms of therapy resistance.

Although the mechanisms of resistance to epidrugs are significantly understudied in GBM compared to other malignancies (especially breast and prostate cancers), some examples can be found in the literature. De Vries and colleagues described the onset of resistance of GBM cells to *Ezh2* knockdown [137]. In their murine models, short-term *Ezh2* knockdown extended survival more robustly than long-term *Ezh2* knockdown did compared to the controls. The authors showed that long-term *Ezh2* knockdown tumours activated a signature of pluripotency genes that might constitute an adaptation mechanism. 

A parallel intrinsic mechanism of resistance to epigenetic therapy could also lie in intratumoural epigenetic heterogeneity in GBM. Several reports based on imaging, chromatin studies, and single-cell RNA-seq and single-cell ATAC-seq clearly showed that cells with a range of chromatin and transcriptional profiles coexist in the same tumours [30,131,138,139,140]. This level of heterogeneity may represent different epigenetic dependencies of subclonal populations, potentially posing challenges to epigenetic therapies. This concept was well-illustrated by work from the Rich laboratory [141]. They showed that transcriptionally proneural GSCs activate EZH2, whereas mesenchymal GSCs activate PRC1 subunit BMI1. Elegant in vitro and in vivo studies demonstrated the need to target both cell populations with concomitant EZH2 and BMI1 inhibition to achieve improved outcomes. 

This work illustrated the importance of the deployment of cocktails of epidrugs to treat GBM. We envision the design of cocktails of epidrugs that maximize the effects on the crucial cellular compartments in a tumour. Given that genetics and epigenetics may interface to predispose genetic subclones to acquire stemlike characteristics [80], genetic and epigenetic assays could be combined to determine the optimal cocktail of epidrugs to use, possibly in a way that is tailored to each patient. A limited number of compounds could be selected on the basis of paradigms of essentiality for epigenetic mechanisms.

Another important consideration pertaining mechanisms of therapeutic resistance is that some epigenetic and chromatin factors play roles outside of the nucleus. For instance, EZH2 can localize in the nucleoli, where it regulates translation [142]. Some epigenetic factors may also have noncanonical functions that should be taken into account when designing epigenetic therapies. For instance, valproic acid has widespread effects outside of the nucleus [143]. EZH2 plays a well-established role in methylating STAT3 specifically in GSCs, resulting in the activation of this transcription factor [144]. The extranuclear and noncanonical functions of epigenetic factors must be considered to fully assess the potential effects of epigenetic therapies in the context of each tumour type.

## 11. Epigenetic Therapy for GBM in the Clinic

Some epigenetic therapies reached the clinic, and we list a few of them in Table 2. This list is not meant to be exhaustive, but it is aimed at highlighting the range of epigenetic and chromatin factors and mechanisms that are being targeted in the patient population.

Several clinical trials are exploring DNA demethylating agents for the treatment of GBM. One of them (clinicaltrials.gov identifier NCT03684811) is a Phase 1b/2 trial that combines methylation inhibitor azacitidine with FT-2102, an inhibitor of mutant IDH1 (Table 2). A second trial employed an oral version of azacitidine (CC-486) in a Phase 1 trial (NCT02223052). Although more work is needed in preclinical models of GBM to better assess the promise of these compounds in the context of this malignancy, several clinical trials are currently active. 

Some clinical trials employed new compounds that target transcription factors. A Phase 1/2 trial (NCT04478279) is testing ST101 (Table 2), a peptide inhibitor of CAAT enhancer binding protein (CEBPB) in GBM and other solid tumours. CEBPB is a positive regulator of mesenchymal-like transcriptional programs, and its knockdown strongly suppresses the engraftment of glioma cells in mice [145]. Furthermore, CEPBP targets are upregulated by radiation treatment [146], suggesting that this protein and its associated transcriptional programs might be involved in therapy resistance pathways. ST101 was granted fast track designation for GBM by the FDA in December 2021.

Another interesting clinical trial from a theoretical perspective tested BRD2/3/4 inhibitor borabresib (MK-8628) in Phase 2 (NCT02296476; Table 2). Preclinical studies demonstrated the ability of BRD4 inhibitors to reduce self-renewal properties of GBM cells both in vitro and in vivo [147,148,149]. Although the clinical trial of borabresib is supported by preclinical evidence, it was recently terminated for lack of clinical activity. This outcome reflects major difficulties in translating even promising discoveries from the lab to the clinic for this highly intractable cancer.

## 12. Concluding Remarks

The pharmaceutical targeting of targets associated with epigenetic and chromatin factors in GSCs is promising for several reasons. First, because epigenomic mechanisms are essential for the maintenance of stemness programs. Second, because epidrugs have the potential to function independently of cell cycle status, an important consideration, given that the most primitive stemlike GBM cells appear to be quiescent or slow cycling. A caveat here is that some epigenetic marks, including histone marks, are stable and require a few cycles of cell division to be sufficiently diluted following the inhibition of the enzyme responsible for their deposition. However, it is critical to eliminate the quiescent GSC compartment to keep the tumour in check. 

An important step in the development of effective epigenetic therapies is the use of relevant and robust preclinical models. At the very least, epidrugs should be tested against patient-derived xenografts in an orthotopic setting. One criticism of these xenograft models is that they are often transplanted in severely immunocompromised hosts which lack the proper tumour microenvironment. These models, therefore, do not fully recapitulate the full repertoire of signalling between neoplastic and nonneoplastic cells or the appropriate metabolic signatures, both being factors that modulate the epigenome of tumour cells. However, xenograft models provide information on the effects of candidate therapies on tumour-cell-intrinsic epigenetic dynamics. Furthermore, they the permit evaluation of pharmacokinetic and physiological measurements of an epidrug in a multicellular organism, providing data that could be very useful in the design of future clinical trials.

Given the wide repertoire of epigenetic pathways regulating programmes of stemness in cancer, it is likely that epidrugs may have to be combined in cocktails to achieve significant therapeutic benefit. This is not unlike nonepigenetic therapeutic cocktails that have been extremely effective in other malignancies. It is highly unlikely that a single compound could be successful in treating a heterogeneous cancer such as GBM.

## Figures and Tables

**Table 1:** Epigenetic factors dysregulated in GBM and compounds targeting them.

Epigenetic Factors	Tool Compounds	Clinically Available Drugs	Reference
PRC2/EZH2	UNC1999, GSK343	Tazemetostat	[110,111]
DNA methylation		5-Azacytidine, decitabine	[116] (review)
WDR5	C16		[126]
Menin/MLL1	MI-2, MI-3	SNDX-5613, KO-539	[128,129,130]
DOT1L		Pinometostat	[134,135]

**Table 2:** Clinical trials are testing epigenetic therapies against a wide range of targets.

Compound	Mode of Action	Other Treatments	Phase	Clinical Trial Number
Azacitidine	DNA methylation inhibitor	FT-2102	1b/2	NCT03684811
CC-486 (oral azacitidine)	DNA methylation inhibitor	N/A	1	NCT02223052
ST101	CEBPB inhibitor	N/A	1/2	NCT04478279
Borabresib (MK-8628)	BRD2/3/4 inhibitor	N/A	2	NCT02296476

## References

[1] Johnstone C.P., Wang N.B., Sevier S.A., Galloway K.E. (2020). Understanding and Engineering Chromatin as a Dynamical System across Length and Timescales. Cell Syst..

[2] Bannister A.J., Kouzarides T. (2011). Regulation of Chromatin by Histone Modifications. Cell Res..

[3] Moore L.D., Le T., Fan G. (2013). DNA Methylation and Its Basic Function. Neuropsychopharmacology.

[4] Statello L., Guo C.-J., Chen L.-L., Huarte M. (2021). Gene Regulation by Long Non-Coding RNAs and Its Biological Functions. Nat. Rev. Mol. Cell Biol..

[5] Verhaak R.G.W., Hoadley K.A., Purdom E., Wang V., Qi Y., Wilkerson M.D., Miller C.R., Ding L., Golub T., Mesirov J.P. (2010). Integrated Genomic Analysis Identifies Clinically Relevant Subtypes of Glioblastoma Characterized by Abnormalities in *PDGFRA*, *IDH1*, *EGFR*, and *NF1*. Cancer Cell.

[6] Wu G., Broniscer A., McEachron T.A., Lu C., Paugh B.S., Becksfort J., Qu C., Ding L., Huether R., Parker M. (2012). Somatic Histone H3 Alterations in Pediatric Diffuse Intrinsic Pontine Gliomas and Non-Brainstem Glioblastomas. Nat. Genet..

[7] Schwartzentruber J., Korshunov A., Liu X.Y., Jones D.T.W., Pfaff E., Jacob K., Sturm D., Fontebasso A.M., Quang D.A.K., Tönjes M. (2012). Driver Mutations in Histone H3.3 and Chromatin Remodelling Genes in Paediatric Glioblastoma. Nature.

[8] Brennan C.W., Verhaak R.G.W., McKenna A., Campos B., Noushmehr H., Salama S.R., Zheng S., Chakravarty D., Sanborn J.Z., Berman S.H. (2013). The Somatic Genomic Landscape of Glioblastoma. Cell.

[9] Pardal R., Clarke M.F., Morrison S.J. (2003). Applying the Principles of Stem-Cell Biology to Cancer. Nat. Rev. Cancer.

[10] Singh S.K., Hawkins C., Clarke I.D., Squire J.A., Bayani J., Hide T., Henkelman R.M., Cusimano M.D., Dirks P.B., Terasaki M. (2003). Identification of a Cancer Stem Cell in Human Brain Tumors. Cancer Res..

[11] Singh S.K., Hawkins C., Clarke I.D., Squire J.A., Bayani J., Hide T., Henkelman R.M., Cusimano M.D., Dirks P.B. (2004). Identification of Human Brain Tumour Initiating Cells. Nature.

[12] Stupp R., Mason W., van den Bent M.J., Weller M., Fisher B.M., Taphoorn M.J.B., Belanger K., Brandes A.A., Marosi C., Bogdahn U. (2005). Radiotherapy plus Concomitant and Adjuvant Temozolomide for Glioblastoma. N. Engl. J. Med..

[13] Poon M.T.C., Sudlow C.L.M., Figueroa J.D., Brennan P.M. (2020). Longer-Term (≥2 Years) Survival in Patients with Glioblastoma in Population-Based Studies Pre- and Post-2005: A Systematic Review and Meta-Analysis. Sci. Rep..

[14] Bao S., Wu Q., Hjelmeland A.B., Rich J.N., Dewhirst M.W., Shi Q., Hao Y., McLendon R.E., Bigner D.D. (2006). Glioma Stem Cells Promote Radioresistance by Preferential Activation of the DNA Damage Response. Nature.

[15] Vlashi E., Lagadec C., Vergnes L., Matsutani T., Masui K., Poulou M., Popescu R., Della Donna L., Evers P., Dekmezian C. (2011). Metabolic State of Glioma Stem Cells and Nontumorigenic Cells. Proc. Natl. Acad. Sci. USA.

[16] Soeda A., Park M., Lee D., Mintz A., Androutsellis-Theotokis A., McKay R.D., Engh J., Iwama T., Kunisada T., Kassam A.B. (2009). Hypoxia Promotes Expansion of the CD133-Positive Glioma Stem Cells through Activation of HIF-1alpha. Oncogene.

[17] Li Z., Bao S., Wu Q., Wang H., Eyler C., Sathornsumetee S., Shi Q., Cao Y., Lathia J., McLendon R.E. (2009). Hypoxia-Inducible Factors Regulate Tumorigenic Capacity of Glioma Stem Cells. Cancer Cell.

[18] Chen J., Li Y., Yu T.-S., McKay R.M., Burns D.K., Kernie S.G., Parada L.F. (2012). A Restricted Cell Population Propagates Glioblastoma Growth after Chemotherapy. Nature.

[19] Xie X.P., Laks D.R., Sun D., Ganbold M., Wang Z., Pedraza A.M., Bale T., Tabar V., Brennan C., Zhou X. (2022). Quiescent Human Glioblastoma Cancer Stem Cells Drive Tumor Initiation, Expansion, and Recurrence Following Chemotherapy. Dev. Cell.

[20] Antonica F., Santomaso L., Pernici D., Petrucci L., Aiello G., Cutarelli A., Conti L., Romanel A., Miele E., Tebaldi T. (2022). A Slow-Cycling/Quiescent Cells Subpopulation Is Involved in Glioma Invasiveness. Nat. Commun..

[21] van Velthoven C.T.J., Rando T.A. (2019). Stem Cell Quiescence: Dynamism, Restraint, and Cellular Idling. Cell Stem Cell.

[22] Codega P., Silva-Vargas V., Paul A., Maldonado-Soto A.R., Deleo A.M., Pastrana E., Doetsch F. (2014). Prospective Identification and Purification of Quiescent Adult Neural Stem Cells from Their in Vivo Niche. Neuron.

[23] Jones K.M., Sarić N., Russell J.P., Andoniadou C.L., Scambler P.J., Basson M.A. (2015). CHD7 Maintains Neural Stem Cell Quiescence and Prevents Premature Stem Cell Depletion in the Adult Hippocampus. Stem Cells.

[24] Llorens-Bobadilla E., Zhao S., Baser A., Saiz-Castro G., Zwadlo K., Martin-Villalba A. (2015). Single-Cell Transcriptomics Reveals a Population of Dormant Neural Stem Cells That Become Activated upon Brain Injury. Cell Stem Cell.

[25] Ziebell F., Dehler S., Martin-Villalba A., Marciniak-Czochra A. (2018). Revealing Age-Related Changes of Adult Hippocampal Neurogenesis Using Mathematical Models. Development.

[26] Bonitto K., Sarathy K., Atai K., Mitra M., Coller H.A. (2021). Is There a Histone Code for Cellular Quiescence?. Front. Cell Dev. Biol..

[27] Bernstein B.E., Mikkelsen T.S., Xie X., Kamal M., Huebert D.J., Cuff J., Fry B., Meissner A., Wernig M., Plath K. (2006). A Bivalent Chromatin Structure Marks Key Developmental Genes in Embryonic Stem Cells. Cell.

[28] Mikkelsen T.S., Ku M., Jaffe D.B., Issac B., Lieberman E., Giannoukos G., Alvarez P., Brockman W., Kim T.-K., Koche R.P. (2007). Genome-Wide Maps of Chromatin State in Pluripotent and Lineage-Committed Cells. Nature.

[29] Cui K., Zang C., Roh T.-Y., Schones D.E., Childs R.W., Peng W., Zhao K. (2009). Chromatin Signatures in Multipotent Human Hematopoietic Stem Cells Indicate the Fate of Bivalent Genes during Differentiation. Cell Stem Cell.

[30] Patel A.P., Tirosh I., Trombetta J.J., Shalek A.K., Gillespie S.M., Wakimoto H., Cahill D.P., Nahed B.V., Curry W.T., Martuza R.L. (2014). Single-Cell RNA-Seq Highlights Intratumoral Heterogeneity in Primary Glioblastoma. Science.

[31] Liau B.B., Sievers C., Donohue L.K., Gillespie S.M., Flavahan W.A., Miller T.E., Venteicher A.S., Hebert C.H., Carey C.D., Rodig S.J. (2017). Adaptive Chromatin Remodeling Drives Glioblastoma Stem Cell Plasticity and Drug Tolerance. Cell Stem Cell.

[32] Burgold T., Spreafico F., De Santa F., Totaro M.G., Prosperini E., Natoli G., Testa G. (2008). The Histone H3 Lysine 27-Specific Demethylase Jmjd3 Is Required for Neural Commitment. PLoS ONE.

[33] Kidder B.L., Hu G., Zhao K. (2014). KDM5B Focuses H3K4 Methylation near Promoters and Enhancers during Embryonic Stem Cell Self-Renewal and Differentiation. Genome Biol..

[34] Tang Q.-Y., Zhang S.-F., Dai S.-K., Liu C., Wang Y.-Y., Du H.-Z., Teng Z.-Q., Liu C.-M. (2020). UTX Regulates Human Neural Differentiation and Dendritic Morphology by Resolving Bivalent Promoters. Stem Cell Rep..

[35] Hamed A.A., Kunz D.J., El-Hamamy I., Trinh Q.M., Subedar O.D., Richards L.M., Foltz W., Bullivant G., Ware M., Vladoiu M.C. (2022). A Brain Precursor Atlas Reveals the Acquisition of Developmental-like States in Adult Cerebral Tumours. Nat. Commun..

[36] Shen T., Cai L.-D., Liu Y.-H., Li S., Gan W.-J., Li X.-M., Wang J.-R., Guo P.-D., Zhou Q., Lu X.-X. (2018). Ube2v1-Mediated Ubiquitination and Degradation of Sirt1 Promotes Metastasis of Colorectal Cancer by Epigenetically Suppressing Autophagy. J. Hematol. Oncol..

[37] Pessoa Rodrigues C., Akhtar A. (2021). Differential H4K16ac Levels Ensure a Balance between Quiescence and Activation in Hematopoietic Stem Cells. Sci. Adv..

[38] Ruthenburg A.J., Li H., Milne T.A., Dewell S., McGinty R.K., Yuen M., Ueberheide B., Dou Y., Muir T.W., Patel D.J. (2011). Recognition of a Mononucleosomal Histone Modification Pattern by BPTF via Multivalent Interactions. Cell.

[39] Xu B., Cai L., Butler J.M., Chen D., Lu X., Allison D.F., Lu R., Rafii S., Parker J.S., Zheng D. (2018). The Chromatin Remodeler BPTF Activates a Stemness Gene-Expression Program Essential for the Maintenance of Adult Hematopoietic Stem Cells. Stem Cell Rep..

[40] Koludrovic D., Laurette P., Strub T., Keime C., Le Coz M., Coassolo S., Mengus G., Larue L., Davidson I. (2015). Chromatin-Remodelling Complex NURF Is Essential for Differentiation of Adult Melanocyte Stem Cells. PLoS Genet..

[41] Green A.L., DeSisto J., Flannery P., Lemma R., Knox A., Lemieux M., Sanford B., O’Rourke R., Ramkissoon S., Jones K. (2020). BPTF Regulates Growth of Adult and Pediatric High-Grade Glioma through the MYC Pathway. Oncogene.

[42] Lee D.H., Kim G.W., Yoo J., Lee S.W., Jeon Y.H., Kim S.Y., Kang H.G., Kim D.-H., Chun K.-H., Choi J. (2021). Histone Demethylase KDM4C Controls Tumorigenesis of Glioblastoma by Epigenetically Regulating P53 and C-Myc. Cell Death Dis..

[43] Mallm J.-P., Windisch P., Biran A., Gal Z., Schumacher S., Glass R., Herold-Mende C., Meshorer E., Barbus M., Rippe K. (2020). Glioblastoma Initiating Cells Are Sensitive to Histone Demethylase Inhibition Due to Epigenetic Deregulation. Int. J. Cancer.

[44] Yan H., Parsons D.W., Jin G., McLendon R., Rasheed B.A., Yuan W., Kos I., Batinic-Haberle I., Jones S., Riggins G.J. (2009). IDH1 and IDH2 Mutations in Gliomas. N. Engl. J. Med..

[45] Zhao S., Lin Y., Xu W., Jiang W., Zha Z., Wang P., Yu W., Li Z., Gong L., Peng Y. (2009). Glioma-Derived Mutations in IDH1 Dominantly Inhibit IDH1 Catalytic Activity and Induce HIF-1alpha. Science.

[46] Dang L., White D.W., Gross S., Bennett B.D., Bittinger M.A., Driggers E.M., Fantin V.R., Jang H.G., Jin S., Keenan M.C. (2009). Cancer-Associated IDH1 Mutations Produce 2-Hydroxyglutarate. Nature.

[47] Tsukada Y., Fang J., Erdjument-Bromage H., Warren M.E., Borchers C.H., Tempst P., Zhang Y. (2006). Histone Demethylation by a Family of JmjC Domain-Containing Proteins. Nature.

[48] Tahiliani M., Koh K.P., Shen Y., Pastor W.A., Bandukwala H., Brudno Y., Agarwal S., Iyer L.M., Liu D.R., Aravind L. (2009). Conversion of 5-Methylcytosine to 5-Hydroxymethylcytosine in Mammalian DNA by MLL Partner TET1. Science.

[49] Carey B.W., Finley L.W.S., Cross J.R., Allis C.D., Thompson C.B. (2015). Intracellular α-Ketoglutarate Maintains the Pluripotency of Embryonic Stem Cells. Nature.

[50] TeSlaa T., Chaikovsky A.C., Lipchina I., Escobar S.L., Hochedlinger K., Huang J., Graeber T.G., Braas D., Teitell M.A. (2016). α-Ketoglutarate Accelerates the Initial Differentiation of Primed Human Pluripotent Stem Cells. Cell Metab..

[51] Chowdhury R., Yeoh K.K., Tian Y.-M., Hillringhaus L., Bagg E.A., Rose N.R., Leung I.K.H., Li X.S., Woon E.C.Y., Yang M. (2011). The Oncometabolite 2-Hydroxyglutarate Inhibits Histone Lysine Demethylases. EMBO Rep..

[52] Lu C., Ward P.S., Kapoor G.S., Rohle D., Turcan S., Abdel-Wahab O., Edwards C.R., Khanin R., Figueroa M.E., Melnick A. (2012). IDH Mutation Impairs Histone Demethylation and Results in a Block to Cell Differentiation. Nature.

[53] Andrade J., Shi C., Costa A.S.H., Choi J., Kim J., Doddaballapur A., Sugino T., Ong Y.T., Castro M., Zimmermann B. (2021). Control of Endothelial Quiescence by FOXO-Regulated Metabolites. Nat. Cell Biol..

[54] Paik J., Ding Z., Narurkar R., Ramkissoon S., Muller F., Kamoun W.S., Chae S.-S., Zheng H., Ying H., Mahoney J. (2009). FoxOs Cooperatively Regulate Diverse Pathways Governing Neural Stem Cell Homeostasis. Cell Stem Cell.

[55] Bulstrode H., Johnstone E., Marques-Torrejon M.A., Ferguson K.M., Bressan R.B., Blin C., Grant V., Gogolok S., Gangoso E., Gagrica S. (2017). Elevated FOXG1 and SOX2 in Glioblastoma Enforces Neural Stem Cell Identity through Transcriptional Control of Cell Cycle and Epigenetic Regulators. Genes Dev..

[56] Kusi M., Zand M., Lin L.-L., Chen M., Lopez A., Lin C.-L., Wang C.-M., Lucio N.D., Kirma N.B., Ruan J. (2022). 2-Hydroxyglutarate Destabilizes Chromatin Regulatory Landscape and Lineage Fidelity to Promote Cellular Heterogeneity. Cell Rep..

[57] Khalil A.M., Guttman M., Huarte M., Garber M., Raj A., Rivea Morales D., Thomas K., Presser A., Bernstein B.E., van Oudenaarden A. (2009). Many Human Large Intergenic Noncoding RNAs Associate with Chromatin-Modifying Complexes and Affect Gene Expression. Proc. Natl. Acad. Sci. USA.

[58] Zhang G., Lan Y., Xie A., Shi J., Zhao H., Xu L., Zhu S., Luo T., Zhao T., Xiao Y. (2019). Comprehensive Analysis of Long Noncoding RNA (LncRNA)-Chromatin Interactions Reveals LncRNA Functions Dependent on Binding Diverse Regulatory Elements. J. Biol. Chem..

[59] Zhang P., Wu W., Chen Q., Chen M. (2019). Non-Coding RNAs and Their Integrated Networks. J. Integr. Bioinform..

[60] Zhao J., Sun B.K., Erwin J.A., Song J.-J., Lee J.T. (2008). Polycomb Proteins Targeted by a Short Repeat RNA to the Mouse X Chromosome. Science.

[61] Rinn J.L., Kertesz M., Wang J.K., Squazzo S.L., Xu X., Brugmann S.A., Goodnough L.H., Helms J.A., Farnham P.J., Segal E. (2007). Functional Demarcation of Active and Silent Chromatin Domains in Human HOX Loci by Noncoding RNAs. Cell.

[62] Venkatraman A., He X.C., Thorvaldsen J.L., Sugimura R., Perry J.M., Tao F., Zhao M., Christenson M.K., Sanchez R., Yu J.Y. (2013). Maternal Imprinting at the *H19-Igf2* Locus Maintains Adult Haematopoietic Stem Cell Quiescence. Nature.

[63] Monnier P., Martinet C., Pontis J., Stancheva I., Ait-Si-Ali S., Dandolo L. (2013). *H19* LncRNA Controls Gene Expression of the Imprinted Gene Network by Recruiting MBD1. Proc. Natl. Acad. Sci. USA.

[64] Cheng Z., Li Z., Ma K., Li X., Tian N., Duan J., Xiao X., Wang Y. (2017). Long Non-Coding RNA XIST Promotes Glioma Tumorigenicity and Angiogenesis by Acting as a Molecular Sponge of MiR-429. J. Cancer.

[65] Jia P., Cai H., Liu X., Chen J., Ma J., Wang P., Liu Y., Zheng J., Xue Y. (2016). Long Non-Coding RNA H19 Regulates Glioma Angiogenesis and the Biological Behavior of Glioma-Associated Endothelial Cells by Inhibiting MicroRNA-29a. Cancer Lett..

[66] Zhang K., Sun X., Zhou X., Han L., Chen L., Shi Z., Zhang A., Ye M., Wang Q., Liu C. (2015). Long Non-Coding RNA HOTAIR Promotes Glioblastoma Cell Cycle Progression in an EZH2 Dependent Manner. Oncotarget.

[67] Yadav B., Pal S., Rubstov Y., Goel A., Garg M., Pavlyukov M., Pandey A.K. (2021). LncRNAs Associated with Glioblastoma: From Transcriptional Noise to Novel Regulators with a Promising Role in Therapeutics. Mol. Ther. Nucleic Acids.

[68] Liang Y., Diehn M., Watson N., Bollen A.W., Aldape K.D., Nicholas M.K., Lamborn K.R., Berger M.S., Botstein D., Brown P.O. (2005). Gene Expression Profiling Reveals Molecularly and Clinically Distinct Subtypes of Glioblastoma Multiforme. Proc. Natl. Acad. Sci. USA.

[69] Sottoriva A., Spiteri I., Piccirillo S.G.M., Touloumis A., Collins V.P., Marioni J.C., Curtis C., Watts C., Tavaré S. (2013). Intratumor Heterogeneity in Human Glioblastoma Reflects Cancer Evolutionary Dynamics. Proc. Natl. Acad. Sci. USA.

[70] Johnson B.E., Mazor T., Hong C., Barnes M., Aihara K., McLean C.Y., Fouse S.D., Yamamoto S., Ueda H., Tatsuno K. (2014). Mutational Analysis Reveals the Origin and Therapy-Driven Evolution of Recurrent Glioma. Science.

[71] Snuderl M., Fazlollahi L., Le L.P., Nitta M., Zhelyazkova B.H., Davidson C.J., Akhavanfard S., Cahill D.P., Aldape K.D., Betensky R.A. (2011). Mosaic Amplification of Multiple Receptor Tyrosine Kinase Genes in Glioblastoma. Cancer Cell.

[72] Little S.E., Popov S., Jury A., Bax D.A., Doey L., Al-Sarraj S., Jurgensmeier J.M., Jones C. (2012). Receptor Tyrosine Kinase Genes Amplified in Glioblastoma Exhibit a Mutual Exclusivity in Variable Proportions Reflective of Individual Tumor Heterogeneity. Cancer Res..

[73] Szerlip N.J., Pedraza A., Chakravarty D., Azim M., McGuire J., Fang Y., Ozawa T., Holland E.C., Huse J.T., Jhanwar S. (2012). Intratumoral Heterogeneity of Receptor Tyrosine Kinases EGFR and PDGFRA Amplification in Glioblastoma Defines Subpopulations with Distinct Growth Factor Response. Proc. Natl. Acad. Sci. USA.

[74] Greaves M., Maley C.C. (2012). Clonal Evolution in Cancer. Nature.

[75] Jordan C.T., Guzman M.L., Noble M. (2006). Cancer Stem Cells. N. Engl. J. Med..

[76] Salgia R., Kulkarni P. (2018). The Genetic/Non-Genetic Duality of Drug “Resistance” in Cancer. Trends Cancer.

[77] Sharma S.V., Lee D.Y., Li B., Quinlan M.P., Takahashi F., Maheswaran S., McDermott U., Azizian N., Zou L., Fischbach M.A. (2010). A Chromatin-Mediated Reversible Drug-Tolerant State in Cancer Cell Subpopulations. Cell.

[78] Eyler C.E., Matsunaga H., Hovestadt V., Vantine S.J., van Galen P., Bernstein B.E. (2020). Single-Cell Lineage Analysis Reveals Genetic and Epigenetic Interplay in Glioblastoma Drug Resistance. Genome Biol..

[79] Neftel C., Laffy J., Filbin M.G., Hara T., Shore M.E., Rahme G.J., Richman A.R., Silverbush D., Shaw M.L., Hebert C.M. (2019). An Integrative Model of Cellular States, Plasticity, and Genetics for Glioblastoma. Cell.

[80] Nikolic A., Singhal D., Ellestad K., Johnston M., Shen Y., Gillmor A., Morrissy S., Cairncross J.G., Jones S., Lupien M. (2021). Copy-ScAT: Deconvoluting Single-Cell Chromatin Accessibility of Genetic Subclones in Cancer. Sci. Adv..

[81] Mazor T., Pankov A., Johnson B.E., Hong C., Hamilton E.G., Bell R.J.A., Smirnov I.V., Reis G.F., Phillips J.J., Barnes M.J. (2015). DNA Methylation and Somatic Mutations Converge on the Cell Cycle and Define Similar Evolutionary Histories in Brain Tumors. Cancer Cell.

[82] Zhu T.S., Costello M.A., Talsma C.E., Flack C.G., Crowley J.G., Hamm L.L., He X., Hervey-Jumper S.L., Heth J.A., Muraszko K.M. (2011). Endothelial Cells Create a Stem Cell Niche in Glioblastoma by Providing NOTCH Ligands That Nurture Self-Renewal of Cancer Stem-like Cells. Cancer Res..

[83] Heddleston J.M., Wu Q., Rivera M., Minhas S., Lathia J.D., Sloan A.E., Iliopoulos O., Hjelmeland A.B., Rich J.N. (2012). Hypoxia-Induced Mixed-Lineage Leukemia 1 Regulates Glioma Stem Cell Tumorigenic Potential. Cell Death Differ..

[84] Heddleston J.M., Li Z., McLendon R.E., Hjelmeland A.B., Rich J.N. (2009). The Hypoxic Microenvironment Maintains Glioblastoma Stem Cells and Promotes Reprogramming towards a Cancer Stem Cell Phenotype. Cell Cycle.

[85] Brooks L.J., Clements M.P., Burden J.J., Kocher D., Richards L., Devesa S.C., Zakka L., Woodberry M., Ellis M., Jaunmuktane Z. (2021). The White Matter Is a Pro-Differentiative Niche for Glioblastoma. Nat. Commun..

[86] Johnson K.C., Anderson K.J., Courtois E.T., Gujar A.D., Barthel F.P., Varn F.S., Luo D., Seignon M., Yi E., Kim H. (2021). Single-Cell Multimodal Glioma Analyses Identify Epigenetic Regulators of Cellular Plasticity and Environmental Stress Response. Nat. Genet..

[87] Ravi V.M., Will P., Kueckelhaus J., Sun N., Joseph K., Salié H., Vollmer L., Kuliesiute U., von Ehr J., Benotmane J.K. (2022). Spatially Resolved Multi-Omics Deciphers Bidirectional Tumor-Host Interdependence in Glioblastoma. Cancer Cell.

[88] Wang Q., Hu B., Hu X., Kim H., Squatrito M., Scarpace L., DeCarvalho A.C., Lyu S., Li P., Li Y. (2017). Tumor Evolution of Glioma-Intrinsic Gene Expression Subtypes Associates with Immunological Changes in the Microenvironment. Cancer Cell.

[89] Varn F.S., Johnson K.C., Martinek J., Huse J.T., Nasrallah M.P., Wesseling P., Cooper L.A.D., Malta T.M., Wade T.E., Sabedot T.S. (2022). Glioma Progression Is Shaped by Genetic Evolution and Microenvironment Interactions. Cell.

[90] Pombo Antunes A.R., Scheyltjens I., Duerinck J., Neyns B., Movahedi K., Van Ginderachter J.A. (2020). Understanding the Glioblastoma Immune Microenvironment as Basis for the Development of New Immunotherapeutic Strategies. Elife.

[91] Zhou W., Ke S.Q., Huang Z., Flavahan W., Fang X., Paul J., Wu L., Sloan A.E., McLendon R.E., Li X. (2015). Periostin Secreted by Glioblastoma Stem Cells Recruits M2 Tumour-Associated Macrophages and Promotes Malignant Growth. Nat. Cell Biol..

[92] Hara T., Chanoch-Myers R., Mathewson N.D., Myskiw C., Atta L., Bussema L., Eichhorn S.W., Greenwald A.C., Kinker G.S., Rodman C. (2021). Interactions between Cancer Cells and Immune Cells Drive Transitions to Mesenchymal-like States in Glioblastoma. Cancer Cell.

[93] Mathewson N.D., Ashenberg O., Tirosh I., Gritsch S., Perez E.M., Marx S., Jerby-Arnon L., Chanoch-Myers R., Hara T., Richman A.R. (2021). Inhibitory CD161 Receptor Identified in Glioma-Infiltrating T Cells by Single-Cell Analysis. Cell.

[94] Babikir H., Wang L., Shamardani K., Catalan F., Sudhir S., Aghi M.K., Raleigh D.R., Phillips J.J., Diaz A.A. (2021). ATRX Regulates Glial Identity and the Tumor Microenvironment in IDH-Mutant Glioma. Genome Biol..

[95] Gangoso E., Southgate B., Bradley L., Rus S., Galvez-Cancino F., McGivern N., Güç E., Kapourani C.-A., Byron A., Ferguson K.M. (2021). Glioblastomas Acquire Myeloid-Affiliated Transcriptional Programs via Epigenetic Immunoediting to Elicit Immune Evasion. Cell.

[96] Parmigiani E., Ivanek R., Rolando C., Hafen K., Turchinovich G., Lehmann F.M., Gerber A., Brkic S., Frank S., Meyer S.C. (2022). Interferon-γ Resistance and Immune Evasion in Glioma Develop via Notch-Regulated Co-Evolution of Malignant and Immune Cells. Dev. Cell.

[97] Venkatesh H.S., Johung T.B., Caretti V., Noll A., Tang Y., Nagaraja S., Gibson E.M., Mount C.W., Polepalli J., Mitra S.S. (2015). Neuronal Activity Promotes Glioma Growth through Neuroligin-3 Secretion. Cell.

[98] Venkatesh H.S., Tam L.T., Woo P.J., Lennon J., Nagaraja S., Gillespie S.M., Ni J., Duveau D.Y., Morris P.J., Zhao J.J. (2017). Targeting Neuronal Activity-Regulated Neuroligin-3 Dependency in High-Grade Glioma. Nature.

[99] Venkatesh H.S., Morishita W., Geraghty A.C., Silverbush D., Gillespie S.M., Arzt M., Tam L.T., Espenel C., Ponnuswami A., Ni L. (2019). Electrical and Synaptic Integration of Glioma into Neural Circuits. Nature.

[100] Venkataramani V., Tanev D.I., Strahle C., Studier-Fischer A., Fankhauser L., Kessler T., Körber C., Kardorff M., Ratliff M., Xie R. (2019). Glutamatergic Synaptic Input to Glioma Cells Drives Brain Tumour Progression. Nature.

[101] Larson J.D., Kasper L.H., Paugh B.S., Jin H., Wu G., Kwon C.H., Fan Y., Shaw T.I., Silveira A.B., Qu C. (2019). Histone H3.3 K27M Accelerates Spontaneous Brainstem Glioma and Drives Restricted Changes in Bivalent Gene Expression. Cancer Cell.

[102] Arrowsmith C.H., Bountra C., Fish P.V., Lee K., Schapira M. (2012). Epigenetic Protein Families: A New Frontier for Drug Discovery. Nat. Rev. Drug Discov..

[103] Scheer S., Ackloo S., Medina T.S., Schapira M., Li F., Ward J.A., Lewis A.M., Northrop J.P., Richardson P.L., Kaniskan H.Ü. (2019). A Chemical Biology Toolbox to Study Protein Methyltransferases and Epigenetic Signaling. Nat. Commun..

[104] Liu J., Perumal N.B., Oldfield C.J., Su E.W., Uversky V.N., Dunker A.K. (2006). Intrinsic Disorder in Transcription Factors. Biochemistry.

[105] Wheeler R.J. (2020). Therapeutics-How to Treat Phase Separation-Associated Diseases. Emerg. Top. Life Sci..

[106] Han X., Yu D., Gu R., Jia Y., Wang Q., Jaganathan A., Yang X., Yu M., Babault N., Zhao C. (2020). Roles of the BRD4 Short Isoform in Phase Separation and Active Gene Transcription. Nat. Struct. Mol. Biol..

[107] Sabari B.R., Dall’Agnese A., Boija A., Klein I.A., Coffey E.L., Shrinivas K., Abraham B.J., Hannett N.M., Zamudio A.V., Manteiga J.C. (2018). Coactivator Condensation at Super-Enhancers Links Phase Separation and Gene Control. Science.

[108] Klein I.A., Boija A., Afeyan L.K., Hawken S.W., Fan M., Dall’Agnese A., Oksuz O., Henninger J.E., Shrinivas K., Sabari B.R. (2020). Partitioning of Cancer Therapeutics in Nuclear Condensates. Science.

[109] Triarico S., Maurizi P., Mastrangelo S., Attinà G., Capozza M.A., Ruggiero A. (2019). Improving the Brain Delivery of Chemotherapeutic Drugs in Childhood Brain Tumors. Cancers.

[110] Stazi G., Taglieri L., Nicolai A., Romanelli A., Fioravanti R., Morrone S., Sabatino M., Ragno R., Taurone S., Nebbioso M. (2019). Dissecting the Role of Novel EZH2 Inhibitors in Primary Glioblastoma Cell Cultures: Effects on Proliferation, Epithelial-Mesenchymal Transition, Migration, and on the pro-Inflammatory Phenotype. Clin. Epigenet..

[111] Qi L., Lindsay H., Kogiso M., Du Y., Braun F.K., Zhang H., Guo L., Zhao S., Injac S.G., Baxter P.A. (2022). Evaluation of an EZH2 Inhibitor in Patient-Derived Orthotopic Xenograft Models of Pediatric Brain Tumors Alone and in Combination with Chemo- and Radiation Therapies. Lab. Investig..

[112] Yu T., Wang Y., Hu Q., Wu W., Wu Y., Wei W., Han D., You Y., Lin N., Liu N. (2017). The EZH2 Inhibitor GSK343 Suppresses Cancer Stem-like Phenotypes and Reverses Mesenchymal Transition in Glioma Cells. Oncotarget.

[113] Del Moral-Morales A., González-Orozco J.C., Hernández-Vega A.M., Hernández-Ortega K., Peña-Gutiérrez K.M., Camacho-Arroyo I. (2022). EZH2 Mediates Proliferation, Migration, and Invasion Promoted by Estradiol in Human Glioblastoma Cells. Front. Endocrinol..

[114] Yin Y., Qiu S., Li X., Huang B., Xu Y., Peng Y. (2017). EZH2 Suppression in Glioblastoma Shifts Microglia toward M1 Phenotype in Tumor Microenvironment. J. Neuroinflamm..

[115] De La Rosa J., Urdiciain A., Zazpe I., Zelaya M.V., Meléndez B., Rey J.A., Idoate M.A., Castresana J.S. (2020). The Synergistic Effect of DZ-NEP, Panobinostat and Temozolomide Reduces Clonogenicity and Induces Apoptosis in Glioblastoma Cells. Int. J. Oncol..

[116] Leone G., Teofili L., Voso M.T., Lübbert M. (2002). DNA Methylation and Demethylating Drugs in Myelodysplastic Syndromes and Secondary Leukemias. Haematologica.

[117] Roulois D., Loo Yau H., Singhania R., Wang Y., Danesh A., Shen S.Y., Han H., Liang G., Jones P.A., Pugh T.J. (2015). DNA-Demethylating Agents Target Colorectal Cancer Cells by Inducing Viral Mimicry by Endogenous Transcripts. Cell.

[118] Chiappinelli K.B., Strissel P.L., Desrichard A., Li H., Henke C., Akman B., Hein A., Rote N.S., Cope L.M., Snyder A. (2015). Inhibiting DNA Methylation Causes an Interferon Response in Cancer via DsRNA Including Endogenous Retroviruses. Cell.

[119] Mehdipour P., Murphy T., De Carvalho D.D. (2020). The Role of DNA-Demethylating Agents in Cancer Therapy. Pharmacol. Ther..

[120] Yamashita A.S., da Costa Rosa M., Borodovsky A., Festuccia W.T., Chan T., Riggins G.J. (2019). Demethylation and Epigenetic Modification with 5-Azacytidine Reduces IDH1 Mutant Glioma Growth in Combination with Temozolomide. Neuro. Oncol..

[121] Turcan S., Rohle D., Goenka A., Walsh L.A., Fang F., Yilmaz E., Campos C., Fabius A.W.M., Lu C., Ward P.S. (2012). IDH1 Mutation Is Sufficient to Establish the Glioma Hypermethylator Phenotype. Nature.

[122] Purow B. (2016). Repurposing Existing Agents as Adjunct Therapies for Glioblastoma. Neuro-Oncol. Pract..

[123] Gallo M., Ho J., Coutinho F., Vanner R., Lee L., Head R., Ling E., Clarke I., Dirks P. (2013). A Tumorigenic MLL-Homeobox Network in Human Glioblastoma Stem Cells. Cancer Res..

[124] Song J.-J., Kingston R.E. (2008). WDR5 Interacts with Mixed Lineage Leukemia (MLL) Protein via the Histone H3-Binding Pocket. J. Biol. Chem..

[125] Southall S.M., Wong P.-S., Odho Z., Roe S.M., Wilson J.R. (2009). Structural Basis for the Requirement of Additional Factors for MLL1 SET Domain Activity and Recognition of Epigenetic Marks. Mol. Cell.

[126] Mitchell K., Shakya S., Silver D.J., Goins C.M., Wallace L., Roversi G., Schafer R., Kay K., Miller T.E., Lauko A. (2021). The WRAD Complex Represents a Therapeutically Exploitable Target for Cancer Stem Cells in Glioblastoma. bioRxiv.

[127] Caslini C., Yang Z., El-Osta M., Milne T.A., Slany R.K., Hess J.L. (2007). Interaction of MLL Amino Terminal Sequences with Menin Is Required for Transformation. Cancer Res..

[128] Borkin D., He S., Miao H., Kempinska K., Pollock J., Chase J., Purohit T., Malik B., Zhao T., Wang J. (2015). Pharmacologic Inhibition of the Menin-MLL Interaction Blocks Progression of MLL Leukemia in Vivo. Cancer Cell.

[129] Grembecka J., He S., Shi A., Purohit T., Muntean A.G., Sorenson R.J., Showalter H.D., Murai M.J., Belcher A.M., Hartley T. (2012). Menin-MLL Inhibitors Reverse Oncogenic Activity of MLL Fusion Proteins in Leukemia. Nat. Chem. Biol..

[130] Krivtsov A.V., Evans K., Gadrey J.Y., Eschle B.K., Hatton C., Uckelmann H.J., Ross K.N., Perner F., Olsen S.N., Pritchard T. (2019). A Menin-MLL Inhibitor Induces Specific Chromatin Changes and Eradicates Disease in Models of MLL-Rearranged Leukemia. Cancer Cell.

[131] Gallo M., Coutinho F.J., Vanner R.J., Gayden T., Mack S.C., Murison A., Remke M., Li R., Takayama N., Desai K. (2015). MLL5 Orchestrates a Cancer Self-Renewal State by Repressing the Histone Variant H3.3 and Globally Reorganizing Chromatin. Cancer Cell.

[132] Lan X., Jörg D.J., Cavalli F.M.G., Richards L.M., Nguyen L.V., Vanner R.J., Guilhamon P., Lee L., Kushida M.M., Pellacani D. (2017). Fate Mapping of Human Glioblastoma Reveals an Invariant Stem Cell Hierarchy. Nature.

[133] Wood K., Tellier M., Murphy S. (2018). DOT1L and H3K79 Methylation in Transcription and Genomic Stability. Biomolecules.

[134] Miller T.E., Liau B.B., Wallace L.C., Morton A.R., Xie Q., Dixit D., Factor D.C., Kim L.J.Y., Morrow J.J., Wu Q. (2017). Transcription Elongation Factors Represent in Vivo Cancer Dependencies in Glioblastoma. Nature.

[135] MacLeod G., Bozek D.A., Rajakulendran N., Monteiro V., Ahmadi M., Steinhart Z., Kushida M.M., Yu H., Coutinho F.J., Cavalli F.M.G. (2019). Genome-Wide CRISPR-Cas9 Screens Expose Genetic Vulnerabilities and Mechanisms of Temozolomide Sensitivity in Glioblastoma Stem Cells. Cell Rep..

[136] Stupp R., Hegi M.E., Mason W.P., van den Bent M.J., Taphoorn M.J., Janzer R.C., Ludwin S.K., Allgeier A., Fisher B., Belanger K. (2009). Effects of Radiotherapy with Concomitant and Adjuvant Temozolomide versus Radiotherapy Alone on Survival in Glioblastoma in a Randomised Phase III Study: 5-Year Analysis of the EORTC-NCIC Trial. Lancet Oncol..

[137] de Vries N.A., Hulsman D., Akhtar W., de Jong J., Miles D.C., Blom M., van Tellingen O., Jonkers J., van Lohuizen M. (2015). Prolonged Ezh2 Depletion in Glioblastoma Causes a Robust Switch in Cell Fate Resulting in Tumor Progression. Cell Rep..

[138] Wang L., Babikir H., Müller S., Yagnik G., Shamardani K., Catalan F., Kohanbash G., Alvarado B., Di Lullo E., Kriegstein A. (2019). The Phenotypes of Proliferating Glioblastoma Cells Reside on a Single Axis of Variation. Cancer Discov..

[139] Richards L.M., Whitley O.K.N., MacLeod G., Cavalli F.M.G., Coutinho F.J., Jaramillo J.E., Svergun N., Riverin M., Croucher D.C., Kushida M. (2021). Gradient of Developmental and Injury Response Transcriptional States Defines Functional Vulnerabilities Underpinning Glioblastoma Heterogeneity. Nat. Cancer.

[140] Guilhamon P., Chesnelong C., Kushida M.M., Nikolic A., Singhal D., MacLeod G., Madani Tonekaboni S.A., Cavalli F.M., Arlidge C., Rajakulendran N. (2021). Single-Cell Chromatin Accessibility Profiling of Glioblastoma Identifies an Invasive Cancer Stem Cell Population Associated with Lower Survival. Elife.

[141] Jin X., Kim L.J.Y., Wu Q., Wallace L.C., Prager B.C., Sanvoranart T., Gimple R.C., Wang X., Mack S.C., Miller T.E. (2017). Targeting Glioma Stem Cells through Combined BMI1 and EZH2 Inhibition. Nat. Med..

[142] Yi Y., Li Y., Meng Q., Li Q., Li F., Lu B., Shen J., Fazli L., Zhao D., Li C. (2021). A PRC2-Independent Function for EZH2 in Regulating RRNA 2’-O Methylation and IRES-Dependent Translation. Nat. Cell Biol..

[143] Ghodke-Puranik Y., Thorn C.F., Lamba J.K., Leeder J.S., Song W., Birnbaum A.K., Altman R.B., Klein T.E. (2013). Valproic Acid Pathway: Pharmacokinetics and Pharmacodynamics. Pharmacogenet. Genom..

[144] Kim E., Kim M., Woo D.H., Shin Y., Shin J., Chang N., Oh Y.T., Kim H., Rheey J., Nakano I. (2013). Phosphorylation of EZH2 Activates STAT3 Signaling via STAT3 Methylation and Promotes Tumorigenicity of Glioblastoma Stem-like Cells. Cancer Cell.

[145] Carro M.S., Lim W.K., Alvarez M.J., Bollo R.J., Zhao X., Snyder E.Y., Sulman E.P., Anne S.L., Doetsch F., Colman H. (2010). The Transcriptional Network for Mesenchymal Transformation of Brain Tumours. Nature.

[146] Halliday J., Helmy K., Pattwell S.S., Pitter K.L., LaPlant Q., Ozawa T., Holland E.C. (2014). In Vivo Radiation Response of Proneural Glioma Characterized by Protective P53 Transcriptional Program and Proneural-Mesenchymal Shift. Proc. Natl. Acad. Sci. USA.

[147] Tao Z., Li X., Wang H., Chen G., Feng Z., Wu Y., Yin H., Zhao G., Deng Z., Zhao C. (2020). BRD4 Regulates Self-Renewal Ability and Tumorigenicity of Glioma-Initiating Cells by Enrichment in the Notch1 Promoter Region. Clin. Transl. Med..

[148] Gusyatiner O., Bady P., Pham M.D.T., Lei Y., Park J., Daniel R.T., Delorenzi M., Hegi M.E. (2021). BET Inhibitors Repress Expression of Interferon-Stimulated Genes and Synergize with HDAC Inhibitors in Glioblastoma. Neuro. Oncol..

[149] Wang Q., Jia S., Wang D., Chen X., Kalvakolanu D.V., Zheng H., Wei X., Wen N., Liang H., Guo B. (2020). A Combination of BRD4 and HDAC3 Inhibitors Synergistically Suppresses Glioma Stem Cell Growth by Blocking GLI1/IL6/STAT3 Signaling Axis. Mol. Cancer Ther..

